# DIVERSE ENVIRONMENTAL CONTEXTS AND LINKS TO LATER-LIFE WELL-BEING

**DOI:** 10.1093/geroni/igad104.0170

**Published:** 2023-12-21

**Authors:** Noah Webster, Markus Schafer

## Abstract

The places older adults spend time have profound impacts on their well-being. As new sources of data and methods become available it is imperative to refine work in this area to guide development of interventions with the precision necessary to address persistent disparities in well-being. This symposium brings together four complementary papers that focus on diverse environmental contexts and well-being using multiple methodological approaches. Perzynski, Berg, and Dalton discuss the potential of Digital Twin Neighborhoods, i.e., digital replicas of real communities, to address health inequalities. They present findings from engagement sessions with community members about priorities, preferences, and concerns with regard to use of digital twins. Sol, Clarke and Zahodne link data from the Detroit-area Michigan Cognitive Aging Project with data from the National Neighborhood Data Archive to examine the association between neighborhood disadvantage and cognitive reserve. They show how this association differs between Black and White participants. Cho, Dunkle, and Smith use data from the Health and Retirement Study (HRS) to examine the association between later life relocation and contact frequency with adult children. They illustrate the importance of proximity and mode of contact in this association. Morris and colleagues also use HRS data to examine the extent to which stressors operating at multiple levels (interpersonal, community, and society) explain racial disparities in memory. They show these contexts together explain 11% of the racial disparity in baseline memory. These papers will be discussed by Markus Schafer who will provide an outlook for future research in this area.

